# Developing a quantitative estimate of muscle age acceleration by a novel phenotypic clock: cross-sectional study in healthy, middle-aged and older adults

**DOI:** 10.18632/aging.206269

**Published:** 2025-06-09

**Authors:** Lucia Ventura, Antonella Cano, Marco Morrone, Gianluca Martinez, Anna Boi, Maria Grazia Catte, Beniamina Mercante, Nicola Loi, Oksana Yurchyshyn, Giovanni Fiorito, Giuliana Solinas, Sara Cruciani, Donatella Coradduzza, Margherita Maioli, Angelo Zinellu, Ciriaco Carru, Franca Deriu, Andrea Manca

**Affiliations:** 1 Department of Biomedical Sciences, University of Sassari, Sassari, Italy; 2 Azienda Ospedaliero-Universitaria di Sassari, Sassari, Italy; 3 Ternopil National Medical University, Ternopil, Ukraine; 4 IRCCS Ospedale Pediatrico Giannina Gaslini, Genova, Italy; 5 Endocrinology Unit, AOUSS, Sassari, Italy

**Keywords:** ageing, biological age, EWGSOP, frailty, sarcopenia

## Abstract

Sarcopenia is a progressive disease characterized by reductions in muscle mass strength and physical performance. Among the initiatives launched to increase awareness, the European Working Group on Sarcopenia in Older People (EWGSOP) is considered the most influential. This cross-sectional study was planned to develop, in healthy middle-aged and older adults, a novel predictor of sarcopenia based on the motor-functional and anthropometric tests derived from EWGSOP2, which were the primary outcome measures.

Participants were tested for body composition, physical performance, blood biomarkers, and risk scores for major healthy issues. Muscle Age Acceleration (MAA) was modelled with Elastic Net regression to extract EWGSOP test mostly contributing to the musculoskeletal ageing trajectory.

Two-hundred-fifteen participants were tested (118 women, 97 men; mean age; 66.0±7.3 years). Muscle Age was correlated with chronological age (r = 0.645; *p* < 0.001). Parsimonious modelling extracted TUG (β = 2.93; 2.48 - to −3.51), ASMM (β = −2.23; −2.99 to −1.67) and Handgrip (β = −1.12; −1.70 to −0.42) for men, and TUG (β = 2.69; 1.96 to 4.19), Handgrip (β = −1.27; −1.56 to −0.98), and Six-MWT (β = −1.15; −1.71 to −0.53) for women. According to MAA, three trajectories were identified: accelerated agers displayed higher risk for sarcopenia (19%), as compared to normal (9%; *p* < 0.0001) and decelerated (2%; *p* < 0.0001), paralleled by significant subclinical alterations of haemato-chemical markers in accelerated agers.

MAA could validly identify accelerated agers with higher risk of sarcopenia, whereas PhenoAge detected subclinical haematochemical alterations. Longitudinal studies are needed to appraise the validity of this newly introduced predictor of sarcopenia and verify if accelerated agers are at higher risk for developing sarcopenia.

## INTRODUCTION

Sarcopenia is a progressive, generalised skeletal muscle disease linked to negative health changes that accumulate across the lifespan [1]. It is common among adults of older age, but occurs also earlier in life, leading to increased likelihood of falls, fractures, disability and mortality. Literally meaning “poverty of flesh” [2], sarcopenia is recognized as a broader disorder where reductions in muscle strength, muscle quantity/quality and physical performance are detected. Multifactorial pathogenesis underpins this condition. From a pathophysiological standpoint, endocrine and metabolic abnormalities interact with the low-grade chronic inflammation (i.e., “inflammageing”), that is observed in advanced agers [3, 4], leading to a reduction of protein-synthesis and regeneration, and a parallel pattern of muscle wasting due to increased apoptosis and protein-lysis [5].

While healthcare professionals are now more aware of this condition and can recognise its negative impact on health, such knowledge has not fully translated into clinical practice. Consequently, sarcopenia is still underdiagnosed and undertreated, leading to a considerable financial burden on healthcare systems for the inherent condition of frailty, increased risk for hospitalisation and cost of care during hospitalisation for sarcopenic compared to non-sarcopenic patients [6].

**Figure 1:**
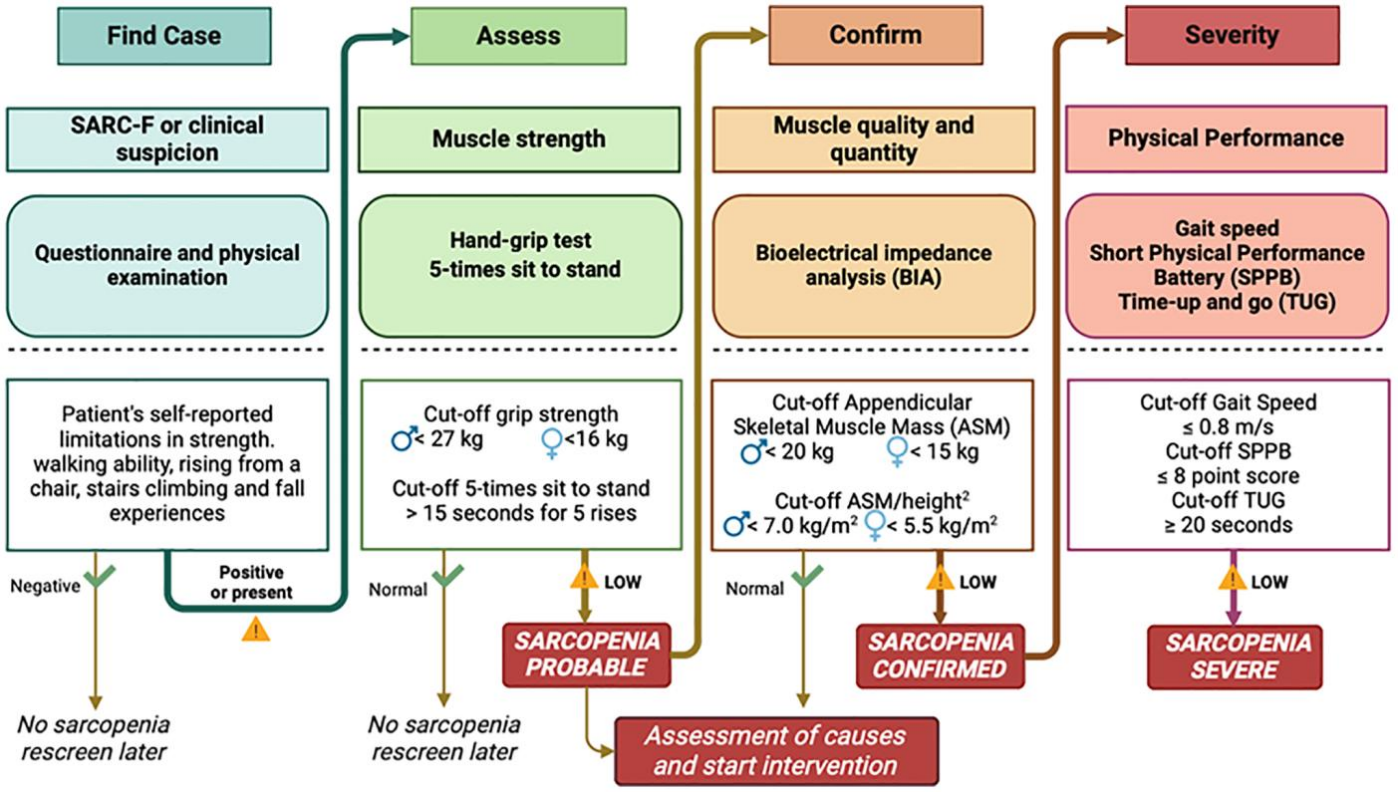
EWGSOP2 algorithm for case-finding, assessment, confirmation and level of severity for sarcopenia (modified from A.J. Cruz-Jentoft et al. *Age and ageing* 2019; 48:16–31) [1].

Among the number of initiatives launched to advance knowledge on sarcopenia and prompt preventive/therapeutic approaches, the European Working Group on Sarcopenia in Older People (EWGSOP) has emerged as the most influential in raising awareness and moving the field forward. EWGSOP consensus firstly introduced a broad clinical definition for sarcopenia not limited to muscle loss [7]. This was eventually developed more recently (EWGSOP2) [1] to move muscle weakness and reduced performance to the forefront as primary indicators of sarcopenia. EWGSOP2's recommendations also developed an algorithm for case-finding, diagnosis, and severity determination for a consistent identification of people with sarcopenia or its risk, and simple, specific cut-off points for measures that identify and characterise sarcopenia. These important implementations are summarised in Figure 1.

While EWSGOP2’s algorithm for sarcopenia screening has undeniably increased awareness of this condition, its categorical nature does not allow to automatically obtain an outcome that quantifies the degree of sarcopenia. Conversely, a scalar, quantitative measure would help identify individuals who do not qualify as sarcopenic despite displaying subclinical alterations that potentially deserve preventive strategies. Such marker would also allow to determine whether interventions aimed at mitigating sarcopenia are truly effective.

This sex-based study, which was conducted in a cohort of healthy, middle-aged and older adults, aimed at (1) developing a predictor of sarcopenia based on the performance of various motor-functional and anthropometric tests, named Muscle Age Acceleration (MAA), as derived from the EWGSOP2 consensus [1], (2) identifying, by means of MAA, subclinical musculoskeletal impairments separately in women and men, and (3) test MAA association with health-related, biological features, including a biological ageing clock built on a set of blood-measured biomarkers strongly predictive of longevity, known as the Phenotypic Age (PhenoAge) [8].

Justification for this study resides in that EWGSOP2-based MAA would allow to capture even subtle, subclinical musculoskeletal dysfunctions also in apparently non-sarcopenic individuals transitioning from middle-age to senescence, allowing predictive selection of individuals at risk for developing sarcopenia.

## RESULTS

Two-hundred-fifteen healthy participants (118 women, 97 men) volunteered for this study. Main demographic, anthropometric, clinical and lifestyle characteristics are summarised in Table 1. Participants were 66.0 ± 7.3 years old (women: 65.7 ± 6.7; men: 66.4 ± 7.8). Women and men were of comparable age (*p* = 0.49) while, as expected, men were significantly heavier and taller (*p* < 0.0001). The two sexes did not differ in terms of median physical activity levels, as estimated by IPAQ (women: 3; men: 3) and comorbidities, as assessed by the median Charlsson Comorbidity Index score (women: 3 ± 0.15; men: 3 ± 0.18).

**Table 1:** Main demographic, anthropometric and psycho-cognitive characteristics of the participants.

	**Total sample (*n* = 215)**	**Women (*n* = 118)**	**Men (*n* = 97)**	**Men vs. Women One-way ANOVA**
**Chronological age (years)**	66.01 ± 7.3	65.7 ± 6.7	66.4 ± 7.8	*p* = 0.487
	(65.1–66.9)	(64.5–66.9)	(64.8–67.9)	
**Weight (kg)**	66.6 ± 12.2	59.1 ± 7.5	76.2 ± 10.0	*p* < 0.0001
	(65.0–68.2)	(57.8–60.4)	(74.3–78.2)	
**Height (cm)**	161.1 ± 10.6	154.1 ± 7.3	170.0 ± 6.9	*p* < 0.0001
	(159.7–162.5)	(152.8–155.4)	(168.7–171.4)	
**Body Mass Index (kg/m^2^)**	25.4 ± 2.8	24.7 ± 2.7	26.3 ± 2.6	*p* < 0.0001
	(25.0–25.8)	(24.3–25.2)	(25.8–26.8)	
**ADL (score)**	6.0 (0.0)	6.0 (0.04)	6.0 (6.04)	*p* = 0.731
	(6.0–8.0)	(6.0–8.0)	(6.0–8.0)	
**I-ADL (score)**	8.0 (0.0)	8.0 (0.0)	8.0 (0.0)	*p* = 0.070
	(8.0–8.0)	(8.0–8.0)	(8.0–8.0)	
**MoCA (adjusted score)**	24.98 (0.24)	24.62 (0.33)	24.98 (0.37)	*p* = 0.594
	(24.6–25.6)	(24.0–25.5)	(24.5–26.0)	
**Charlsson Comorbidity Index (*n*)**	2.78 (1.34)	2.80 (1.35)	2.75 (1.34)	*p* = 0.837
	(2.56–3.01)	(2.50–3.10)	(2.40–3.11)	
**IPAQ (1-low; 2-moderate; 3-high)**	2 (0.06)	2 (0.04)	2 (0.09)	*p* = 0.367
	(1.8–2.2)	(1.7–2.1)	(1.9–2.3)	

All data are presented as mean ± standard deviation and confidence interval of the mean, except for ADL, I-ADL, MoCA scores and IPAQ, which are expressed as median (standard error) and (confidence interval of the median). Abbreviations: kg: kilograms; cm: centimeters; m^2^: square meters; ADL: Activities of Daily Living; I-ADL: Instrumental ADL; MoCA: Montreal Cognitive Assessment.

### Muscle age and muscle age acceleration (MAA)

**Figure 2:**
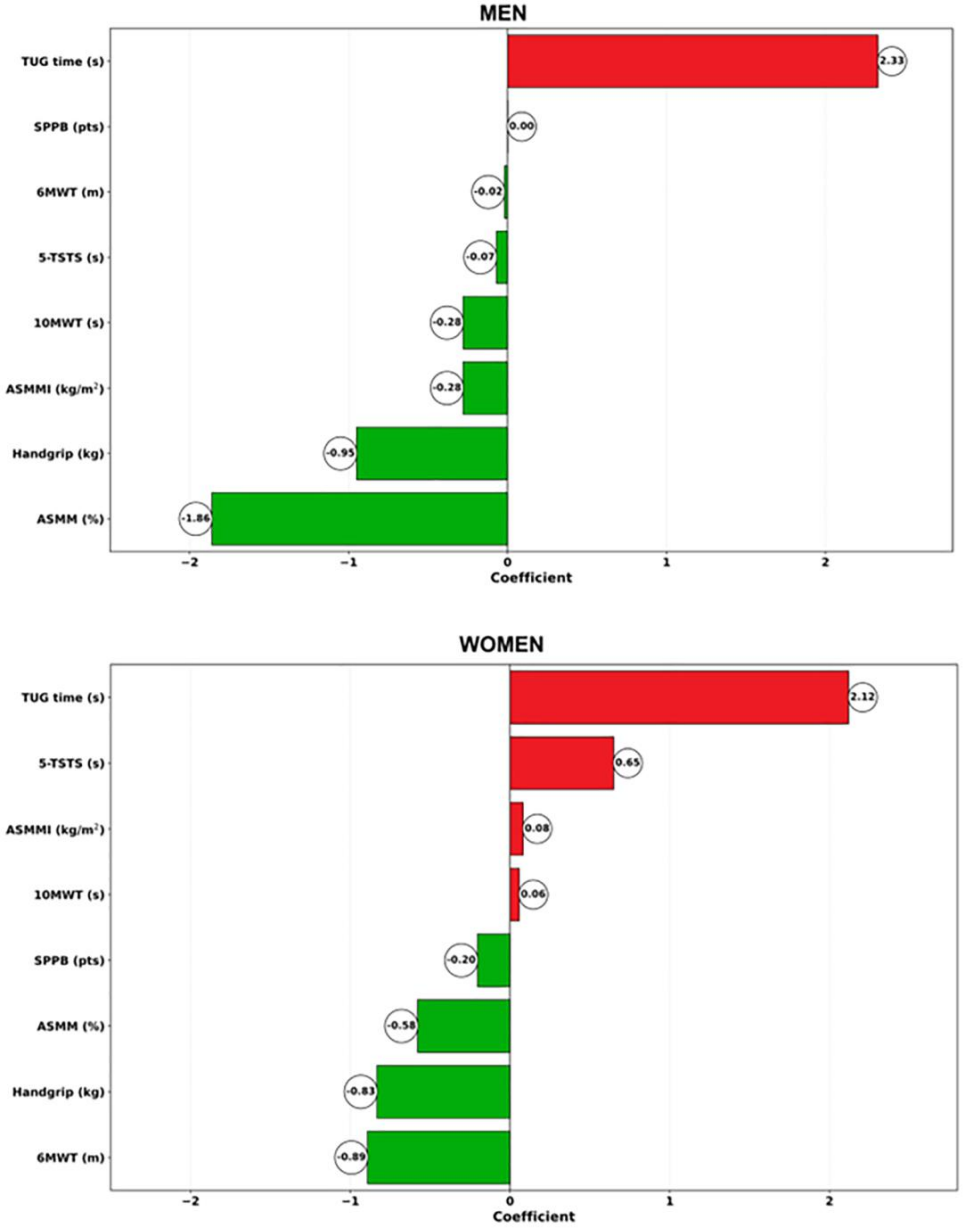
Contributors from the penalized elastic net regression model run with coefficients indicating the standardized weight in women (upper panel) and men (lower panel).

Derived from the penalised elastic net model, Muscle Age was significantly correlated with chronological age (r = 0.645; *p* < 0.001). Figure 2 displays the sex-based contributors based on the results of the motor-functional tests recommended by EWGSOP2. The contributions of each test in accelerating (red bars) or decelerating (green bars) the ageing trajectory are reported as coefficients indicating the standardised weight of each test in the construction of the biological/Muscle Age. Coefficients equal to zero indicate no contribution to the ageing measure. Being standardised, the weights can be read as the increase in Muscle Age for each increase by one standard deviation of the corresponding test result. Positive coefficients/weights indicate motor tests whose results are higher in an individual with higher Muscle Age and vice versa. Parsimonious modelling to retain only those features mostly contributing to the estimation of MAA extracted TUG (β = 2.93; 2.48- to −3.51), ASMM (β = −2.23; −2.99 to −1.67) and Handgrip (β = −1.12; −1.70 to −0.42) for men, and TUG (β = 2.69; 1.96 to 4.19), Handgrip (β = −1.27; −1.56 to −0.98), and Six-MWT (β = −1.15; −1.71 to −0.53) for women. RMSE and R^2^ of these parsimonious models were RMSE = 5.56 ± 0.90, R^2^ = 0.42 ± 0.17 for men, and RMSE = 5.43±0.65, R^2^ = 0.35 ± 0.16 for women.

Muscle Age was then linearly regressed on chronological age and the residuals retained as age accelerations in years, i.e., MAA. This allowed to account for the inherent age-related decline of motor-functional performance that is positively correlated with age.

### Classifying participants according to MAA

Based on the above results, participants were classified into three categories of agers according to the percentile-based distribution of the data: ‘decelerated’ (up to 25th percentile: *n* = 53; 23W:30M) for individuals with MAA scores lower than −3.8 years for women and −3.3 years for men; ‘normal’ (26th to 75th percentile: *n* = 109; 69W:40M) for individuals with MAA between −3.8 and +2.5 years for women and −3.3 to +4.2 for men; ‘accelerated’ (from 76th percentile: *n* = 53; 26W:27M) for individuals with MAA higher than 2.5 years for women and 4.2 years for men.

### Comparing MAA trajectories

**Figure 3:**
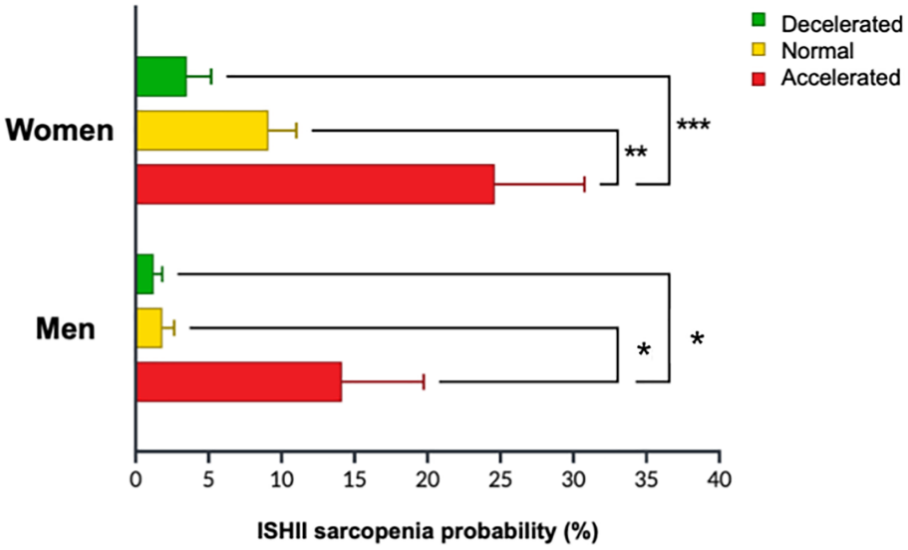
Probability of sarcopenia according to ISHII sarcopenia screening [11] in women and men based on Muscle Age Acceleration (MAA) status.

Comparisons among the three MAA categories carried out by general linear model ANOVA on the whole cohort are detailed in Table 2. MAA could identify participants at higher risk for sarcopenia, as assessed by Ishii’s formula [9], with accelerated agers displaying 19% probability of sarcopenia, which proved significantly higher than normal (9%; *p* < 0.0001) and decelerated agers (2%; *p* < 0.0001). Sex-based comparisons showed higher probability of sarcopenia both in accelerated (14%) compared to normal (2%) and decelerated male agers (all *p*-values ≤ 0.03). The same applied to women (accelerated: 25%; normal: 9%; decelerated: 4%; all *p*-values ≤ 0.001) (Figure 3).

**Table 2:** Descriptive and statistic results by European Working Group of Sarcopenia in Older People (EWGSOP2) assessments according to Muscle Age Acceleration (MAA) status and sex (*N* = 215; 53 decelerated, 109 normal, 53 accelerated agers; 118 women, 97 men).

		**MAA status**			**Statistics**	
		**Decelerated**	**Normal**	**Accelerated**	**Main effect**	**Pairwise comparisons**
**Ishii sarcopenia probability**	Whole sample	0.02 (0.02)	0.05 (0.02)	0.19 (0.03)	MAA status F = 13.79 *p* < 0.0001	Decel<Accel *p* < 0.0001 Normal<Accel *p* < 0.0001
	Women	0.04 (0.04)	0.09 (0.02)	0.25 (0.03)	Sex F = 6.30 *p* = 0.013	Decel<Accel *p* < 0.0001 Normal<Accel *p* < 0.001
	Men	0.01 (0.03)	0.02 (0.03)	0.14 (0.04)	MAA*Sex F = 0.69 *p* = 0.503	Decel<Accel *p* = 0.034 Normal<Accel *p* = 0.030
**Handgrip strength (kg)**	Whole sample	31.66 (0.70)	28.57 (0.52)	25.39 (0.70)	MAA status F = 19.90 *p* < 0.0001	Decel>Accel *p* < 0.0001 Decel>Normal *p* = 0.001 Normal>Accel *p* = 0.001
	Women	23.47 (1.01)	20.68 (0.61)	18.66 (0.96)	Sex F = 413.35 *p* < 0.0001	Decel>Accel *p* = 0.002
	Men	39.85 (0.97)	36.46 (0.84)	32.11 (1.04)	MAA*Sex F = 1.27 *p* = 0.283	Decel>Normal *p* = 0.027 Decel>Accel *p* < 0.0001 Normal>Accel *p* = 0.004
**Five-TSTS (s)**	Whole sample	7.07 (0.22)	7.36 (0.16)	8.68 (0.22)	MAA status F = 16.05 *p* < 0.0001	Decel<Accel *p* < 0.0001 Normal<Accel *p* < 0.0001
	Women	6.71 (0.32)	7.27 (0.19)	8.92 (0.30)	Sex F = 0.36 *p* = 0.549	Decel<Accel *p* < 0.0001 Normal<Accel *p* < 0.0001
	Men	7.42 (0.30)	7.45 (0.26)	8.44 (0.32)	MAA*Sex F = 1.87 *p* = 0.158	ns
**SMM (kg)**	Whole sample	25.19 (0.35)	23.30 (0.26)	22.23 (0.35)	MAA status F = 18.39 *p* < 0.0001	Decel>Normal *p* < 0.0001 Decel>Accel *p* < 0.0001 Normal>Accel *p* = 0.045
	Women	18.95 (0.51)	17.32 (0.30)	16.42 (0.48)	Sex F = 1034.58 *p* < 0.0001	Decel>Normal *p* = 0.019 Decel>Accel *p* = 0.001
	Men	31.42 (0.49)	29.28 (0.42)	28.03 (0.52)	MAA*Sex F = 0.38 *p* = 0.685	Decel>Normal *p* = 0.003 Decel>Accel *p* < 0.0001
**ASMM (kg)**	Whole sample	19.92 (0.29)	18.53 (0.22)	17.72 (0.29)	MAA status F = 14.50 *p* < 0.0001	Decel>Normal *p* = 0.001 Decel>Accel *p* < 0.0001
	Women	15.90 (0.42)	14.87 (0.25)	14.18 (0.40)	Sex F = 573.86 *p* < 0.0001	Decel>Accel *p* = 0.010
	Men	23.93 (0.41)	22.18 (0.35)	21.26 (0.43)	MAA*Sex F = 0.75 *p* = 0.473	Decel>Accel *p* < 0.0001 Decel>Norm *p* = 0.004
***n*-10MWT (m/s)**	Whole sample	1.56 (0.03)	1.52 (0.02)	1.39 (0.03)	MAA status F = 11.52 *p* < 0.0001	Decel>Accel *p* < 0.0001 Normal>Accel *p* < 0.001
	Women	1.60 (0.04)	1.51 (0.02)	1.35 (0.04)	Sex F = 0.008 *p* = 0.929	Decel>Accel *p* < 0.0001 Normal>Accel *p* < 0.001
	Men	1.51 (0.04)	1.53 (0.03)	1.42 (0.04)	MAA*Sex F = 2.35 *p* = 0.098	ns
**SPPB (score)**	Whole sample	11.98 (0.06)	11.90 (0.05)	11.78 (0.06)	MAA status F = 2.77 *p* = 0.065	ns
	Women	11.96 (0.09)	11.87 (0.05)	11.81 (0.08)	Sex F = 0.05 *p* = 0.827	ns
	Men	12.00 (0.09)	11.94 (0.07)	11.74 (0.09)	MAA*Sex F = 0.51 *p* = 0.604	ns
**TUG (s)**	Whole sample	5.82 (0.12)	6.30 (0.09)	7.26 (0.12)	MAA status F = 39.67 *p* < 0.0001	Decel<Accel *p* < 0.001 Decel<Normal *p* = 0.004 Normal<Accel *p* < 0.001
	Women	5.81 (0.17)	6.61 (0.10)	7.56 (0.16)	Sex F = 10.23 *p* = 0.002	Decel<Normal *p* < 0.001 Decel<Accel *p* < 0.0001 Normal<Accel *p* < 0.0001
	Men	5.84 (0.16)	5.99 (0.14)	6.95 (0.17)	MAA*Sex F = 2.81 *p* = 0.062	Decel<Accel *p* < 0.0001 Normal<Accel *p* < 0.0001
**Six-MWT (m)**	Whole sample	561.45 (9.08)	532.00 (6.69)	474.14 (9.10)	MAA status F = 24.21 *p* < 0.0001	Decel>Normal *p* = 0.029 Decel>Accel *p* < 0.0001 Normal>Accel *p* = 0.001
	Women	572.95 (13.10)	508.56 (7.84)	456.39 (12.35)	Sex F = 4.19 *p* = 0.042	Decel>Normal *p* < 0.001 Decel>Accel *p* < 0.0001 Normal>Accel *p* = 0.001
	Men	549.94 (12.58)	555.44 (10.85)	491.88 (13.38)	MAA*Sex F = 4.99 *p* = 0.008	Decel>Accel *p* = 0.005 Normal>Accel *p* = 0.001

All data are scores, which are shown as mean ± standard deviation. Abbreviations: Accel: accelerated agers; Decel: decelerated agers; Normal: normal agers; MAA: Muscle Age Acceleration; kg: kilograms; Five-TSTS: Five-time sit-to-stand; s, seconds; ns: no significance; SMM: Skeletal Muscle Mass; ASMM: Appendicular Skeletal Muscle Mass; n10MWT: 10Meter Walked Test-normal Walk; m: meter; SPPB: Short Physical Performance Battery; TUG: Timed Up and Go Test; Six-MWT: Six-minute Walking Test.

No significant main effects of MAA, sex or interaction term (MAA_status*sex) emerged for the scores of MOCA, ADL, IADL, and health status (total and sub-scores). However, MOCA proved significantly correlated with MAA in the whole group (r = −0.14; *p* = 0.04; *n* = 212) and, more specifically, in the accelerated subgroup (r = −0.39; *p* = 0.03).

Table 3 summarises the main effects of MAA, sex and MAA*sex interaction for the comparisons run on the haematochemical samples. Bonferroni-corrected pairwise comparisons revealed a significant difference in WBC count only for men when comparing decelerated (5.62/μL) vs. accelerated agers (6.63/μL; +18%; *p* = 0.02). Regardless of sex, HGB mass was significantly lower in accelerated (13.5 g/dL) compared to decelerated (14.2 g/dL; *p* < 0.0001) and normal agers (14.0 g/dL; *p* < 0.0001). HCT was significantly lower in accelerated agers (42.7%) compared to normal agers (44.4%; *p* = 0.04). Only among women MCV was found significantly smaller (*p* = 0.02) for accelerated (82.1 μm^3^) compared to decelerated agers (90.3 μm^3^). The same applied to MCH for which women with accelerated ageing showed lower MCH (25.9 μm^3^) than decelerated ones (29.1 pg; *p* = 0.02). %RDW was found significantly higher in accelerated women than decelerated ones (14.32% and 13.50%, respectively; *p* = 0.04).

**Table 3:** Haemato-chemical sample results analysed by Muscle Age Acceleration (MAA) status and sex (*N* = 133, 33 decelerated, 67 normal, 33 decelerated agers; 53 men, 80 women).

		**MAA status**			**Statistics**	
		**Decelerated**	**Normal**	**Accelerated**	**Main effect**	**Pairwise comparisons**
**White Blood Cell (× 10^3^/μL)**	Whole sample	5.46 (0.22)	6.00 (0.18)	6.10 (0.23)	MAA status F = 2.55 *p* = 0.082	ns
	Women	5.29 (0.32)	5.56 (0.18)	5.58 (0.32)	Sex F = 9.74 *p* = 0.002	ns
	Men	5.62 (0.31)	6.44 (0.30)	6.63 (0.32)	MAA*Sex F = 0.74 *p* = 0.480	Decel<Accel *p* = 0.024
**Red Blood Cells (× 10^3^/μL)**	Whole sample	5.05 (0.10)	5.10 (0.08)	5.06 (0.10)	MAA status F = 0.09 *p* = 0.915	ns
	Women	4.70 (0.14)	4.91 (0.08)	5.05 (0.14)	Sex F = 12.17 *p* = 0.001	ns
	Men	5.41 (0.14)	5.29 (0.13)	5.08 (0.14)	MAA*Sex F = 2.99 *p* = 0.054	ns
**Hemoglobin (g/dL)**	Whole sample	14.16 (0.20)	14.19 (0.16)	13.49 (0.21)	MAA status F = 4.16 *p* = 0.018	Decel<Accel *p* < 0.0001 Normal<Accel *p* < 0.0001
	Women	13.56 (0.29)	13.59 (0.17)	12.76 (0.29)	Sex F = 34.10 *p* < 0.0001	Normal>Accel *p* = 0.015
	Men	14.76 (0.28)	14.80 (0.27)	14.21 (0.29)	MAA*Sex F = 0.13 *p* = 0.879	ns
**Haematocrit test (%)**	Whole sample	44.47 (0.54)	44.35 (0.43)	42.66 (0.55)	MAA status F = 3.68 *p* = 0.028	Normal>Accel *p* = 0.048
	Women	42.13 (0.77)	42.60 (0.44)	40.67 (0.77)	Sex F = 48.11 *p* < 0.0001	Normal>Accel *p* = 0.032
	Men	46.80 (0.75)	46.11 (0.73)	44.66 (0.77)	MAA*Sex F = 0.36 *p* = 0.699	Decel>Accel *p* = 0.048
**Mean Corpuscular Volume (μm^3^)**	Whole sample	88.27 (1.78)	87.87 (1.41)	85.48 (1.80)	MAA status F = 0.74 *p* = 0.478	ns
	Women	90.33 (2.55)	87.73 (1.46)	82.08 (2.55)	Sex F = 0.26 *p* = 0.611	Decel>Accel *p* = 0.024
	Men	86.21 (2.47)	88.01 (2.40)	88.88 (2.55)	MAA*Sex F = 2.36 *p* = 0.098	ns
**Mean corpuscular hemoglobin (10 × (Hb/RBC))**	Whole sample	28.23 (0.66)	28.07 (0.52)	27.04 (0.67)	MAA status F = 1.00 *p* = 0.371	ns
	Women	29.11 (0.94)	27.87 (0.54)	25.85 (0.94)	Sex F = 0.23 *p* = 0.632	Decel>Accel *p* = 0.016
	Men	27.35 (0.92)	28.28 (0.89)	28.23 (0.94)	MAA*Sex F = 2.43 *p* = 0.092	ns
**Mean Corpuscular Hemoglobin Concentration (g/dL)**	Whole sample	31.85 (0.19)	31.98 (0.15)	31.60 (0.19)	MAA status F = 1.22 *p* = 0.300	ns
	Women	32.14 (0.27)	31.88 (0.15)	31.41 (0.27)	Sex F = 0.00 *p* = 0.965	ns
	Men	31.55 (0.26)	32.07 (0.25)	31.79 (0.27)	MAA*Sex F = 1.98 *p* = 0.142	ns
**Red Blood Cell Distribution Width (%)**	Whole sample	13.80 (0.19)	13.72 (0.15)	14.06 (0.19)	MAA status F = 0.99 *p* = 0.376	ns
	Women	13.50 (0.28)	13.72 (0.16)	14.32 (0.28)	Sex F = 0.02 *p* = 0.892	Decel<Accel *p* = 0.038
	Men	14.09 (0.27)	13.72 (0.26)	13.81 (0.28)	MAA*Sex F = 2.81 *p* = 0.133	ns
**Hemoglobin distribution width (mg/dL)**	Whole sample	2.50 (0.06)	2.49 (0.05)	2.51 (0.07)	MAA status F = 0.04 *p* = 0.957	ns
	Women	2.37 (0.09)	2.43 (0.05)	2.25 (0.09)	Sex F = 3.00 *p* = 0.086	ns
	Men	2.62 (0.09)	2.55 (0.09)	2.51 (0.09)	MAA*Sex F = 1.06 *p* = 0.350	ns
**Platelets (x 10^9^/L)**	Whole sample	216.07 (8.10)	232.83 (6.41)	226.06 (8.22)	MAA status F = 1.32 *p* = 0.272	ns
	Women	236.31 (11.63)	245.39 (6.65)	232.31 (11.63)	Sex F = 8.75 *p* = 0.004	ns
	Men	195.82 (11.28)	220.28 (10.96)	219.81 (11.63)	MAA*Sex F = 0.74 *p* = 0.480	ns
**Mean Platelet Volume (fL)**	Whole sample	8.18 (0.16)	8.07 (0.12)	8.18 (0.16)	MAA status F = 0.24 *p* = 0.790	ns
	Women	8.24 (0.23)	7.91 (0.13)	8.12 (0.23)	Sex F = 0.37 *p* = 0.543	ns
	Men	8.12 (0.22)	8.23 (0.21)	8.24 (0.23)	MAA*Sex F = 0.61 *p* = 0.546	ns
**Neutrophils (%)**	Whole sample	53.76 (1.43)	55.99 (1.13)	55.00 (1.45)	MAA status F = 0.75 *p* = 0.472	ns
	Women	51.82 (2.05)	53.20 (1.17)	52.91 (2.05)	Sex F = 8.58 *p* = 0.004	ns
	Men	55.69 (1.99)	58.79 (1.949)	57.10 (2.05)	MAA*Sex F = 0.13 *p* = 0.875	ns
**Lymphocytes (%)**	Whole sample	34.42 (1.33)	32.44 (1.05)	32.96 (1.35)	MAA status F = 0.69 *p* = 0.503	ns
	Women	37.12 (1.91)	35.56 (1.09)	34.80 (1.91)	Sex F = 12.42 *p* = 0.001	ns
	Men	31.72 (1.85)	29.33 (1.80)	31.12 (1.91)	MAA*Sex F = 0.28 *p* = 0.757	ns
**Monocytes (%)**	Whole sample	5.99 (0.23)	6.05 (0.18)	6.27 (0.23)	MAA status F = 0.40 *p* = 0.669	ns
	Women	5.62 (0.33)	5.61 (0.19)	6.29 (0.33)	Sex F = 4.29 *p* = 0.040	ns
	Men	6.36 (0.32)	6.48 (0.31)	6.24 (0.33)	MAA*Sex F = 1.30 *p* = 0.277	ns
**Eosinophils (%)**	Whole sample	2.83 (0.29)	2.75 (0.23)	3.27 (0.29)	MAA status F = 1.03 *p* = 0.359	ns
	Women	2.57 (0.41)	2.75 (0.23)	2.84 (0.41)	Sex F = 2.27 *p* = 0.134	ns
	Men	3.10 (0.40)	2.76 (0.39)	3.70 (0.41)	MAA*Sex F = 0.704 *p* = 0.496	ns
**Basophils (%)**	Whole sample	0.69 (0.05)	0.66 (0.04)	0.67 (0.05)	MAA status F = 0.10 *p* = 0.903	ns
	Women	0.68 (0.07)	0.69 (0.04)	0.74 (0.07)	Sex F = 1.38 *p* = 0.242	ns
	Men	0.69 (0.07)	0.63 (0.07)	0.60 (0.07)	MAA*Sex F = 0.597 *p* = 0.552	ns
**Large Unstained Cells (%)**	Whole sample	2.31 (0.13)	2.08 (0.11)	2.47 (0.14)	MAA status F = 2.60 *p* = 0.078	ns
	Women	2.19 (0.19)	2.18 (0.11)	2.44 (0.19)	Sex F = 0.05 *p* = 0.823	ns
	Men	2.42 (0.19)	1.98 (0.18)	2.50 (0.19)	MAA*Sex F = 0.85 *p* = 0.428	ns
**Neutrophils (× 10^3^/μL)**	Whole sample	2.99 (0.16)	3.38 (0.13)	3.36 (0.16)	MAA status F = 2.20 *p* = 0.115	ns
	Women	2.80 (0.23)	2.98 (0.13)	3.01 (0.23)	Sex F = 13.55 *p* < 0.001	ns
	Men	3.17 (0.22)	3.79 (0.21)	3.71 (0.23)	MAA*Sex F = 0.63 *p* = 0.533	Decel<Normal *p* = 0.044
**Lymphocytes (× 10^3^/μL)**	Whole sample	1.85 (0.10)	1.93 (0.08)	1.97 (0.10)	MAA status F = 0.40 *p* = 0.673	ns
	Women	1.93 (0.14)	1.98 (0.08)	1.90 (0.14)	Sex F = 0.11 *p* = 0.74	ns
	Men	1.77 (0.14)	1.89 (0.13)	2.04 (0.14)	MAA*Sex F = 0.60 *p* = 0.548	ns
**Monocytes (× 10^3^/μL)**	Whole sample	0.33 (0.02)	0.35 (0.01)	0.39 (0.02)	MAA status F = 3.69 *p* = 0.028	Decel<Accel *p* = 0.023
	Women	0.29 (0.02)	0.31 (0.01)	0.36 (0.02)	Sex F = 16.25 *p* < *p* < 0.0001	ns
	Men	0.36 (0.02)	0.39 (0.02)	0.42 (0.02)	MAA*Sex F = 0.14 *p* = 0.868	ns
**Eosinophils (× 10^3^/μL)**	Whole sample	0.16 (0.02)	0.17 (0.01)	0.22 (0.02)	MAA status F = 2.89 *p* = 0.059	ns
	Women	0.14 (0.03)	0.15 (0.02)	0.17 (0.03)	Sex F = 6.41 *p* = 0.013	ns
	Men	0.17 (0.03)	0.18 (0.03)	0.26 (0.03)	MAA*Sex F = 1.01 *p* = 0.365	Decel<Accel *p* = 0.015 Normal<Accel *p* = 0.033
**Basophils (x10^3^/μL)**	Whole sample	0.03 (0.02)	0.04 (0.01)	0.04 (0.02)	MAA status F = 0.09 *p* = 0.913	ns
	Women	0.03 (0.02)	0.06 (0.01)	0.04 (0.02)	Sex F = 0.43 *p* = 0.515	ns
	Men	0.04 (0.02)	0.02 (0.02)	0.03 (0.02)	MAA*Sex F = 0.527 *p* = 0.592	ns
**Large Unstained Cells (× 10^3^/μL)**	Whole sample	0.13 (0.01)	0.13 (0.01)	0.16 (0.01)	MAA status F = 2.84 *p* = 0.062	ns
	Women	0.11 (0.02)	0.12 (0.01)	0.14 (0.02)	Sex F = 3.95 *p* = 0.049	ns
	Men	0.14 (0.01)	0.13 (0.01)	0.17 (0.02)	MAA*Sex F = 0.15 *p* = 0.864	ns
**Glycemia (mg/dL)**	Whole sample	94.75 (2.09)	95.17 (1.66)	89.44 (2.09)	MAA status F = 2.57 *p* = 0.081	ns
	Women	93.31 (2.96)	92.98 (1.69)	86.00 (2.96)	Sex F = 4.33 *p* = 0.039	ns
	Men	96.19 (2.96)	97.35 (2.87)	92.88 (2.96)	MAA*Sex F = 0.23 *p* = 0.791	ns
**Urea (mg/dL)**	Whole sample	35.32 (1.64)	37.62 (1.30)	37.53 (1.66)	MAA status F = 0.69 *p* = 0.505	ns
	Women	33.94 (2.35)	35.51 (1.34)	35.75 (2.35)	Sex F = 3.89 *p* = 0.051	ns
	Men	36.71 (2.28)	39.72 (2.22)	39.31 (2.35)	MAA*Sex F = 0.06 *p* = 0.942	ns
**Creatinine (mg/dL)**	Whole sample	0.90 (0.02)	0.88 (0.02)	0.85 (0.03)	MAA status F = 0.874 *p* = 0.422	ns
	Women	0.77 (0.04)	0.77 (0.02)	0.80 (0.04)	Sex F = 48.5 *p* < 0.0001	ns
	Men	1.03 (0.03)	0.99 (0.04)	0.91 (0.04)	MAA*Sex F = 2.60 *p* = 0.083	Decel>Accel *p* = 0.031
**Total protein (g/dL)**	Whole sample	7.08 (0.07)	7.07 (0.06)	6.97 (0.07)	MAA status F = 0.86 *p* = 0.426	ns
	Women	7.06 (0.10)	7.11 (0.06)	6.93 (0.10)	Sex F = 0.08 *p* = 0.782	ns
	Men	7.11 (0.10)	7.03 (0.10)	7.01 (0.10)	MAA*Sex F = 0.45 *p* = 0.641	ns
**Albumin (g/dL)**	Whole sample	4.26 (0.04)	4.27 (0.03)	4.18 (0.04)	MAA status F = 1.73 *p* = 0.182	ns
	Women	4.26 (0.05)	4.26 (0.03)	4.14 (0.05)	Sex F = 0.66 *p* = 0.419	ns
	Men	4.26 (0.05)	4.27 (0.05)	4.23 (0.05)	MAA*Sex F = 0.39 *p* = 0.677	ns
**Alkaline phosphatase (U/L)**	Whole sample	75.65 (3.61)	80.34 (2.86)	73.25 (3.67)	MAA status F = 1.27 *p* = 0.283	ns
	Women	79.25 (5.18)	74.79 (2.96)	76.12 (5.18)	Sex F = 0.02 *p* = 0.883	ns
	Men	72.06 (5.03)	85.94 (4.89)	70.38 (5.18)	MAA*Sex F = 2.66 *p* = 0.074	ns
**Serum iron (μg/dL)**	Whole sample	98.19 (4.81)	90.83 (3.89)	91.94 (4.89)	MAA status F = 0.76 *p* = 0.472	ns
	Women	91.19 (6.91)	89.59 (3.95)	87.44 (6.91)	Sex F = 2.60 *p* = 0.109	ns
	Men	105.18 (6.70)	92.06 (6.70)	96.44 (6.91)	MAA*Sex F = 0.45 *p* = 0.640	ns
**C-reactive protein (mg/dL)**	Whole sample	0.40 (0.03)	0.38 (0.02)	0.38 (0.03)	MAA status F = 0.30 *p* = 0.738	ns
	Women	0.42 (0.04)	0.35 (0.02)	0.40 (0.04)	Sex F = 0.27 *p* = 0.604	ns
	Men	0.38 (0.04)	0.40 (0.03)	0.35 (0.04)	MAA*Sex F = 1.50 *p* = 0.227	ns
**Interleukin 6 (ng/mL)**	Whole sample	790.75 (46.06)	722.22 (36.45)	756.96 (46.76)	MAA status F = 0.69 *p* = 0.502	ns
	Women	715.26 (66.12)	689.17 (37.78)	694.72 (66.12)	Sex F = 5.18 *p* = 0.025	ns
	Men	866.23 (64.15)	755.28 (62.34)	819.21 (66.12)	MAA*Sex F = 0.29 *p* = 0.750	ns
**Insulin-like growth factor-1 (ng/ml)**	Whole sample	0.76 (0.14)	0.81 (0.12)	0.74 (0.15)	MAA status F = 0.07 *p* = 0.933	ns
	Women	0.98 (0.21)	0.81 (0.12)	0.77 (0.21)	Sex F = 1.20 *p* = 0.276	ns
	Men	0.54 (0.19)	0.80 (0.21)	0.70 (0.22)	MAA*Sex F = 0.75 *p* = 0.476	ns
**Leptin (ng/mL)**	Whole sample	452.10 (60.03)	495.45 (47.50)	374.81 (60.94)	MAA status F = 1.22 *p* = 0.299	ns
	Women	469.38 (86.18)	435.64 (49.24)	442.05 (86.18)	Sex F = 0.06 *p* = 0.801	ns
	Men	434.82 (83.60)	555.26 (81.25)	307.58 (86.18)	MAA*Sex F = 1.44 *p* = 0.242	ns
**Plasma protein-SH (micromol/g/proteins)**	Whole sample	5.09 (0.15)	4.63 (0.12)	4.54 (0.15)	MAA status F = 4.00 *p* = 0.021	Decel>Accel *p* = 0.035
	Women	5.01 (0.22)	4.58 (0.13)	4.44 (0.22)	Sex F = 0.89 *p* = 0.346	ns
	Men	5.17 (0.21)	4.67 (0.20)	4.64 (0.22)	MAA*Sex F = 0.04 *p* = 0.963	ns
**Malondialdehyde (micromol/L)**	Whole sample	2.73 (0.20)	2.73 (0.16)	2.40 (0.21)	MAA status F = 0.93 *p* = 0.398	ns
	Women	2.35 (0.29)	2.32 (0.17)	1.94 (0.29)	Sex F = 13.76 *p* < 0.001	ns
	Men	3.11 (0.28)	3.13 (0.28)	2.85 (0.29)	MAA*Sex F = 0.03 *p* = 0.968	ns
**Paraoxonase (U/L)**	Whole sample	159.09 (15.51)	168.53 (12.34)	129.39 (16.00)	MAA status F = 1.92 *p* = 0.152	ns
	Women	169.78 (22.26)	153.16 (12.99)	131.30 (22.99)	Sex F = 0.01 *p* = 0.914	ns
	Men	148.39 (21.59)	183.90 (20.99)	127.48 (22.26)	MAA*Sex F = 0.93 *p* = 0.394	ns
**Homocysteine (micromol/L)**	Whole sample	1.17 (0.08)	1.04 (0.06)	1.17 (0.08)	MAA status F = 1.15 *p* = 0.318	ns
	Women	1.29 (0.12)	1.03 (0.07)	1.19 (0.11)	Sex F = 1.03 *p* = 0.313	ns
	Men	1.05 (0.11)	1.05 (0.11)	1.15 (0.11)	MAA*Sex F = 0.85 *p* = 0.430	ns

All data are scores, which are shown as mean ± standard deviation. Abbreviations: MAA: Muscle Age Acceleration; μL: microliters; g: grams; ns: no significance; mg/dl: milligrams/deciliters; μm^3^: cubic micrometer; hb/RBC: hemoglobin/Red Blood Cell; L: liters; fL: femtolitreng/mL nanograms/milliliters; U: unit.

Monocyte count was significantly higher in accelerated (3.87/μL) compared to decelerated agers (3.33/μL; *p* = 0.02), with no differences by sex. Conversely, only among men eosinophil count was significantly higher in accelerated (0.26/μL) compared to normal (0.18/μL, *p* = 0.03) and decelerated agers (0.17/μL; *p* = 0.02). Likewise, neutrophil count was significantly lower in decelerated men (3.17/μL) compared to normal (3.79/μL; *p* = 0.04) but not accelerated agers. The same applied to creatinine, which was found significantly higher in decelerated men (1.03 mg/dL) compared to accelerated (0.91 mg/dL; *p* = 0.03). Regardless of sex, plasma PSH was significantly higher in decelerated (5.09 μmol/g) compared to accelerated agers (4.54 μmol/g; *p* = 0.03).

**Figure 4:**
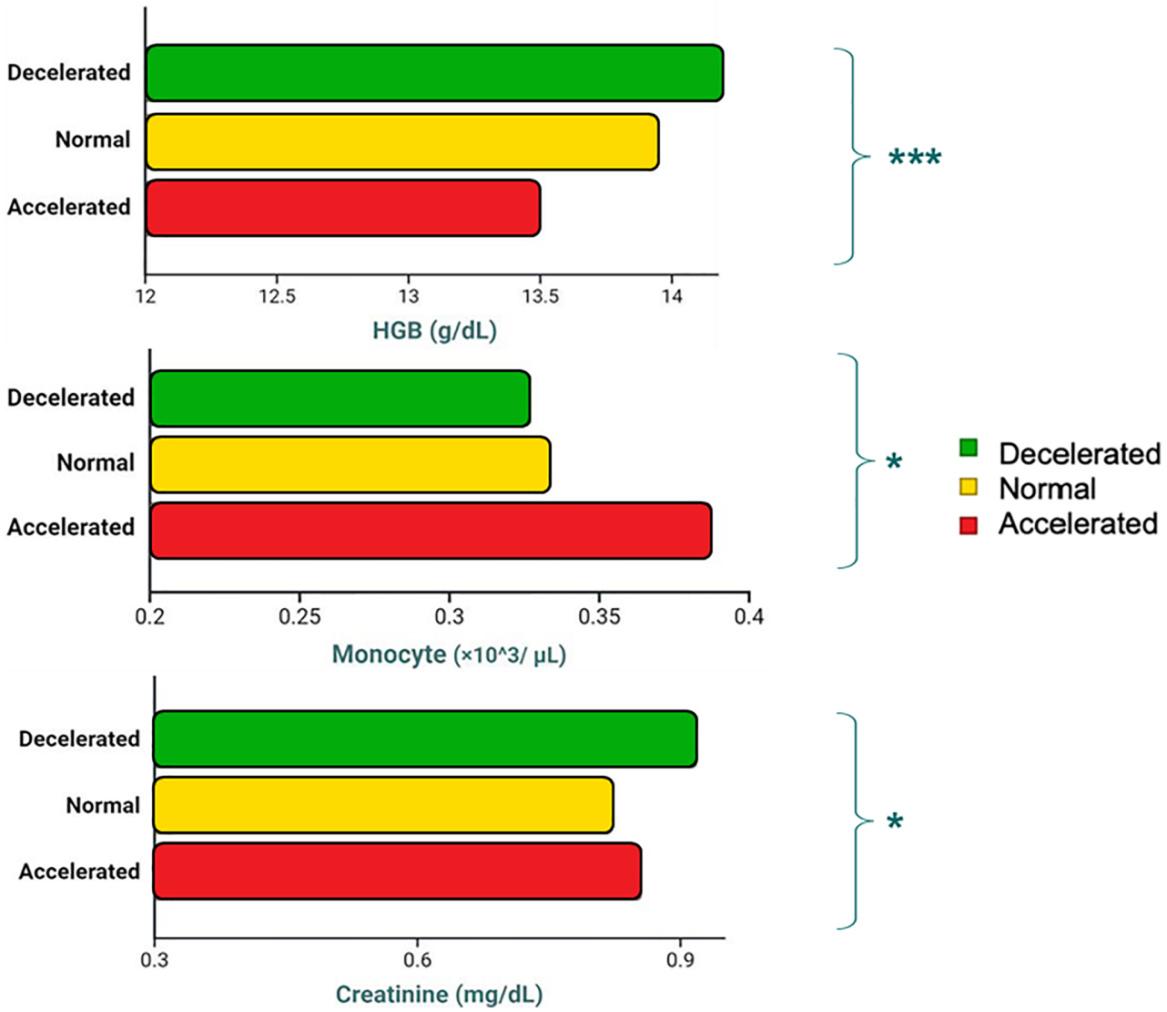
Differences in exemplificative blood-based markers depending on Muscle Age Acceleration (MAA) status (Decelerated vs. Normal vs. Accelerated).

No differences by MAA emerged for RBC, mean corpuscular haemoglobin concentration, haemoglobin distribution width, platelets count, mean platelet volume, neutrophil %, lymphocyte %, monocyte %, eosinophil %, basophil %, large unstained cells %, lymphocyte count, basophil count, large unstained cells count, glycemia, plasma concentration of urea, albumin, total proteins, alkaline phosphatase, serum iron, CRP, IL-6, IGF-1, leptine, malondialdehyde, and paraoxonase, and homocysteine (Table 3). Figure 4 shows a visual comparison of decelerated, normal, and accelerated aging trajectories for three exemplificative haematochemical parameters that proved to significantly differ by MAA category.

### Biological age estimation

The PhenoAge calculator by Levine (2018) [8] allowed to estimate participants’ biological age and the related age acceleration. While Phenotypic and Muscle ages proved significantly correlated with one another (r = 0.59; *p* < 0.001) as well as with chronological age (r = 0.90 and 0.69, respectively; *p* < 0.001), no correlation was found between the derived measures of age acceleration, MAA and biological age acceleration (r = 0.01; *p* = 0.87).

### Health risks

Of the two markers of age acceleration, only PhenoAA proved significantly correlated, in both women and men, with all cardiovascular risk scores and mortality risk, but not with comorbidities (Table 4). Conversely, MAA was associated with mortality risk only in men.

**Table 4:** Correlation matrix of Muscle Age Acceleration and Phenotypic Age Acceleration with health risk scores.

		**MAA**	**PhenoAA**	**FRS**	**ACCAHA**	**STERN**	**MESA**	**CCI**	**Mortality score**
**MAA**	Whole sample	–	0,014	−0,150	−0,032	−0,173	0,027	−0,061	−0,149^*^
	Women	–	0,135	−0,182	−0,123	−0,117	0,008	−0,122	−0,028
	Men	–	−0,120	−0,135	−0,075	−0,179	0,082	0,027	−0,252^*^
**PhenoAA**	Whole sample	0,014	–	0,280^*^	0,220	0,245^**^	0,314^*^	0,004	0,431^**^
	Women	0,135	–	0,067	0,149	0,406^**^	0,167	0,027	0,398^**^
	Men	−0,120	–	−0,120	−0,021	0,148	0,062	−0,034	0,463^**^

Data are described as Spearman’s Rho. ^*^Correlation is significant at the 0.05 level (1-tailed); ^**^Correlation is significant at the 0.01 level (1-tailed). Abbreviations: MAA: Muscle Age Acceleration; PhenoAA: Phenotypic Age Acceleration; FRS: Framingham risk score coronary heart disease; ACCAHA: American College of Cardiology and American Heart Association cardiovascular diseases risk score; STERN: Stern Diabetes Risk; MESA: Multi-Ethnic Study of Atherosclerosis risk score; CCI: Charlson Comorbidity Index.

## DISCUSSION

This study was conducted in healthy, middle-aged and older adults aged 50–90 years, to (1) portray their pattern of musculoskeletal ageing through a comprehensive estimate of sarcopenia, MAA, (2) test the ability of this tool to identify subclinical yet potentially prodromal musculoskeletal dysfunctions, and (3) verify whether MAA associated with health-related, biological features.

To these ends, we employed the screening tools recommended by EWGSOP2 consensus on sarcopenia [1], and the phenotypic clock developed by Levine [8], concurrently assessing morphological, motor-functional and biological markers, as increasingly advocated by researchers and practitioners active in ageing research [5].

In line with our hypothesis, using evidence-based, widely supported anthropometric and motor-functional tests allowed to build a marker of musculoskeletal ageing, which could identify early musculoskeletal ageing in a subgroup of apparently non-sarcopenic individuals. According to our estimates, these accelerated agers (approximately 25% of the cohort) would be at higher risk for developing sarcopenia. Although EWGSOP2 tests individually did not result in detecting overtly sarcopenic individuals, participants who displayed a combination of even slight anthropometric and motor-functional alterations had a 4-to-6-fold higher probability of developing sarcopenia, according to Ishii’s formula [9]. A quantitative assessment would also potentially provide an objective, scalar physiological proxy of musculoskeletal ageing useful to investigate pathophysiological mechanisms subtending sarcopenia and to evaluate the effect of interventions in reversing/slowing this age-related condition [6]. In this regard, since TUG time and handgrip strength were extracted from the whole set of EWGSOP2 tools as major contributors to MAA both in women and men, substantial improvements in these selected tests following, for instance, reconditioning and strengthening exercise, would likely lead to decelerated musculoskeletal ageing. TUG and handgrip have been previously put forward as foremost predictors of independence and short-term mortality in the elderly, and reduced performances in these tests are taken as red flags [10, 11]. Indeed, the values at these tests are considered relevant markers of physical capability and healthy ageing [12] and qualify as practically relevant endpoints of treatment to appraise the effectiveness of preventive/therapeutic approaches. TUG, which implies rising from a chair, accelerating to a target, turn around it and return to the starting position, embodies physical and executive functions that are critical for independence and safety but subjected to substantial age-related decline.

As a preliminary validation of the newly introduced clock of musculoskeletal ageing, we found it strongly associated with PhenoAge, as calculated by the ensemble of blood-based markers contributing to the Levine’s epigenetic clock [8]. However, only the latter correlated in a significant manner to major health risks like coronary and cardiovascular diseases, atherosclerosis, and mortality. This would suggest that these musculoskeletal and biological proxies of ageing process address different health domains that could be comprehensively captured by their concurrent evaluation. This seems particularly interesting from a pathophysiological standpoint as it suggests that the musculoskeletal system may start to decline even when signs of increased health risk are absent or not yet developed. In this context, sarcopenia would follow a distinct, anticipated trajectory, as is often the case with early-onset sarcopenia, which onsets subclinically as soon as fifth or even fourth decade of life. It also points toward what is possibly a closer relationship between lifestyle and musculoskeletal well-being, the latter being more strongly influenced by sedentariness and the inherent lack of mechanical loading than systemic health (as resampled by PhenoAge).

As such, our data point toward a complementary use of the tools here investigated, with MAA serving as a practical, low-cost independent predictor of sarcopenia, and PhenoAge as a blood-based estimate of major health issues, which, as such, are not suited to capture musculoskeletal deterioration. While our data contribute to a growing literature supporting the utility of biological clocks to estimate risk profiles, especially for cardiovascular health, further research is needed to determine the extent to which biological clocks can be used as clinical biomarkers [13].

As a further validation of MAA, once the cohort was classified according to MAA (decelerated/normal/accelerated), several health-related and haemato-chemical scores polarized into a decelerated/healthier pattern as opposed to accelerated/unhealthier, with normal agers generally located among the two clusters yet generally closer to decelerated ones. It nevertheless has to be said that our cohort exhibited haemato-chemical values falling within normative ranges in all domains, even though the subgroup of accelerated agers displayed biological features (e.g., monocyte and eosinophil count, haemoglobin mass, creatinine, etc.) compatible with latent, low-grade inflammation and higher activation of the immune system that are signature elements of overt sarcopenia [14, 15]. Relatedly, given that systemic inflammation plays a key role in muscle aging and considering the bidirectional communication and influence between the gut microbiota (the community of microorganisms living in the intestines) and skeletal muscle, integrating gut microbiome alterations into the discussion could strengthen the argument that muscle aging is not only a localized process but influenced by systemic metabolic and inflammatory factors [16, 17]. Clearly, such factors concur and synergize in the frailty of the elderly, a multidimensional phenomenon involving musculoskeletal decline, systemic aging, and resilience loss [18]. In this framework, MAA may represent a potential predictor and early marker of frailty severity and progression as well as an endpoint of treatment if interventions to mitigate frailty are administered.

Longitudinal profiling of our cohort through follow-up evaluations of musculoskeletal and biological ageing will provide a unique opportunity to further validate MAA through a prevalence study to confirm or reject the hypothesis that accelerated agers have significantly higher likelihood of developing sarcopenia than normal and decelerated agers to evolve from subclinical features to a definite diagnosis of sarcopenia at three-five years from the current screening. Such follow-up re-evaluations with prevalence data will also allow to validate the regression-based predictive equations developed in our cohort, leading to the establishment of straightforward formulae that can be employed to quantify musculoskeletal ageing in research and clinical settings. So far, we have developed preliminary weighted-average equations for estimating the individual Muscle Age based on the parsimonious model of best predictors here extracted (see: Supplementary Material 1).

### Study limitations

The first limitation relates to the population under study, as the present findings were obtained from healthy, non-sarcopenic subjects, who were not compared in terms of musculoskeletal and biological ageing arcs to comparable yet overtly sarcopenic individuals. Upcoming controlled studies are needed to appraise the validity of MAA to differentiate between sarcopenic and non-sarcopenic subjects and capture different degrees of sarcopenia severity. Another weakness relates to the sample over which validation of MAA against biological, haemato-chemical markers could be completed, as approximately 60% of the original cohort underwent both musculoskeletal and biological testing.

Finally, the cross-sectional design chosen for this study cannot dynamically portray the curve of the individual ageing process over a defined timespan, preventing comparisons of predicted versus actual trajectories. To do so, a longitudinal design with repeated follow-up measurements is warranted, also taking potential confounders like (but not limited to) lifestyle, physical activity levels, comorbidities and socioeconomic profile into account. This effort has been initiated and is currently underway for the present cohort. Repeated measurements would also allow to establish the test-retest consistency and responsiveness of MAA by calculating indexes such as intraclass correlation coefficient, standard error of measurement and minimal detectable difference, which would strengthen the reliability of upcoming studies.

## CONCLUSIONS

Non-sarcopenic, community-dwelling middle-aged and older adults could be validly classified in terms of their individual musculoskeletal ageing trajectories with a novel muscular clock, MAA. This comprehensive estimate, developed from the EWGSOP2 framework, could identify signs of accelerated musculoskeletal ageing when subclinical, slight alterations presented concurrently. Participants with such signs were also those displaying slightly altered haemato-chemical features.

If validated through a properly planned, population-based, controlled, longitudinal study, this novel estimate may represent an objective tool for a comprehensive and cost-effective evaluation of the musculoskeletal wellbeing of the elderly. In this perspective, MAA may complement and strengthen other quantitative and qualitative tools for sarcopenia screening including, but not limited to, the Ishii test for sarcopenia [9] and the SARC-F questionnaire [19]. The parsimonious models consisting of three very simple and common motor tests could be easily administered both in clinical and community settings. To this end, the simple formula here provided (see: Results) will allow to estimate MAA on a vast scale, at least for screening purposes, until a more robust validation of this tool is attained, potentially green flagging more diagnostic applications.

## MATERIALS AND METHODS

This cross-sectional study was conducted at the Department of Biomedical Sciences, University of Sassari, Italy, from September 2020 to December 2023. Potential candidates were searched through phone interviews and public engagement initiatives.

Participants were required to be aged 50–90 years with no medical, physical, or cognitive conditions that might interfere with functional assessments. After eligibility screening, a clinical examination assessed exclusion criteria like severe respiratory, neurological, or cardiovascular diseases, recent injuries, and metabolic conditions. The cognitive status was also monitored by Montreal Cognitive Assessment (MOCA), as well as functioning and independence during activities of daily living (ADL) [20] and instrumental ADL (IADL) [21] through questionnaires.

### Sarcopenia assessment

Participants were evaluated according to the EWGSOP2 guidelines using the Find-Assess-Confirm-Severity approach [1]. Sarcopenia was screened with the Ishii test, which calculates risk using an equation combining age, grip strength, and calf circumference. A score >105 for men and >120 for women indicates high probability of sarcopenia [9].

### Muscle strength

Handgrip strength (HGS) was measured using a handgrip dynamometer (G200, Biometrics Ltd., UK) over three trials, with one-minute rests. The 5-times sit-to-stand test (5-TSTS) was performed to estimate lower body strength [22].

### Body composition

Skeletal muscle mass (SMM) and appendicular (ASMM) were assessed via bioelectrical impedance analysis (BIA 101 BIVA; Akern, Italy) [23].

### Physical performance

Gait speed was measured with the 10-Meter Walk Test (10MWT) at a comfortable walking pace [24]. Physical performance was evaluated by Short Physical Performance Battery (SPPB), assessing gait speed, balance, and strength [24]. The Timed Up and Go Test (TUG) was used to assess fall risk, balance and functional mobility [25]. The distance covered during the Six-minute Walk Test (Six-MWT) was recorded to evaluate cardiovascular fitness [26].

### Blood analyses and biomarkers

Blood samples were analysed for several key parameters, including white blood cells (WBC), red blood cells (RBC), haemoglobin (HGB), haematocrit (HCT), creatinine, urea, alkaline phosphatase, serum iron, and C-reactive protein (CRP). Concentrations of interleukin-6 (IL-6), leptin, tumour necrosis factor-alpha (TNF-α), and insulin-like growth factor 1 (IGF-1) were measured by ELISA.

Oxidative stress markers were also assessed, with paraoxonase-1 (PON-1) activity measured using paraoxon as a substrate, protein-SH (PSH) levels, and malondialdehyde (MDA) levels.

Selected blood-measured biomarkers were combined to calculate the PhenoAge, aka biological age, according to Levine [8].

Risk scores for selected major health issues were calculated using the Framingham Risk Score (FRS) for coronary heart disease [27], CVD risk from the American College of Cardiology (ACC) and American Heart Association (AHA) [28], and 10-year risk of atherosclerosis based on the Multi-Ethnic Study of Atherosclerosis (MESA) study [29]. The risk of diabetes 2 [30], 10-year mortality risk score [8], and Charlson Comorbidity Index (CCI) [31] were also calculated.

### Statistical analysis

#### Muscle age and muscle age acceleration modelling

Muscle Age and its derivative measure of acceleration MAA were modelled separately for men and women to account for sex differences in ageing trajectories, using a two-phase approach. First, all tests from EWGSOP2 consensus were used as predictors. Muscle Age was modelled with Elastic Net regression (ElasticNetCV, Scikit-learn v.1.5.1), with 100 permutations and 80/20 train-test splits [32]. Five-fold cross-validation was used to optimize regularisation strength (alpha) and the balance between L1 and L2 penalties (l1_ratio). Average root mean square error (RMSE) of the differences between predicted and observed values and R^2^ of the regression across the 100 permutations were reported as metrics of robustness and stability of the predictive models. Predictor coefficients representing the relative importance of each feature were averaged across permutations for robustness. Secondly, for parsimonious modelling, only those predictors with beta coefficients ≥1 standard deviation were included in refined, sex-specific models, using the same Elastic Net method. Sex-specific models were developed to reflect the established sexual differences in aging that are evident in the early and later phases of adulthood and senescence.

For both phases, MAA was calculated as residuals from the linear regression of predicted versus chronological age. Subjects were then categorised into three groups (MAA_status) based on MAA scores: decelerated (0–25th percentile), normal (26th-75th percentile), and accelerated (above 76th percentile).

Shapiro-Wilk’s, Mauchly’s and Levene’s tests were performed to appraise data normality, sphericity and homogeneity. A general linear model analysis of variance (ANOVA) was conducted to examine the main effects of MAA_status, sex and the interaction MAA_status*sex on functional and health-related outcomes. Pairwise comparisons were adjusted (Bonferroni) to reduce type-I error rate.

Correlation analysis was performed to assess the association between MAA and other continuous variables, including the relationship with PhenoAge acceleration (PhenoAA) which, likewise MAA, was calculated as the residuals from the linear regression of predicted versus chronological age.

### New and noteworthy

This study introduces an evidence-based phenotypic clock, the Muscle Age Acceleration (MAA), which could identify signs of accelerated musculoskeletal ageing when subclinical, slight alterations presented concurrently. Participants with such signs were also those displaying slightly altered haemato-chemical features. If validated through a properly planned, population-based, controlled longitudinal study, this novel predictor of musculoskeletal decline may represent an objective tool for a comprehensive and cost-effective evaluation of sarcopenia for individuals transitioning from middle-age to senescence.

### Data availability

All datasets are stored at the Department of Biomedical Sciences, University of Sassari, Sassari, Italy, under the responsibility of Professor Franca Deriu. Data will be made available upon reasonable request.

## Supplementary Materials

Supplementary Material 1
