# Supplementary material for: Developing a quantitative estimate of muscle age acceleration by a novel phenotypic clock: cross-sectional study in healthy, middle-aged and older adults
Source: Aging (Albany NY). 2025 Jun 9;17(6):1466–83. doi: 10.18632/aging.206269 (PMC12245203; doi:10.18632/aging.206269)
Supplement: Supplementary Material 1 [file aging-17-206269-s001.pdf]

SUPPLEMENTARY MATERIAL 1

Formulae to estimate the individual muscle age*

Men

MuscleAge = 65.65 + 2.92 · Scaled_TUG - 1.13 · Scaled_Handgrip - 2.26 · Scaled_ASMM

where:
Scaled measure = (individual measurement – mean of the study sample)/standard deviation of the study sample
Scaled TUG[#] = (TUG time (s) - 6.04)/1.08
Scaled Handgrip = (Handgrip (kg) - 36.45)/7.15
Scaled ASMM= (ASMM (kg) - 22.6/2.76

Women
MuscleAge = 65.72 - 2.54 · Scaled_TUG - 1.25 · Scaled_Handgrip - 1.21 · Scaled_6MWT

where:
Scaled TUG = (TUG time (s) - 6.69)/1.00
Scaled Handgrip = (Handgrip (kg) - 20.69)/4.17
Scaled 6MWT = (6MWT (m) - 508.18)/74.00

**based on data from 215 moderately active healthy participants (118 women, 97 men; mean age; 66.0±7.3 years)*
[#] for men's TUG, which data were significantly skewed to the left of the distribution curve (skewness = 2.209), median and interquartile range of the study sample by Ventura et al. were used instead of mean and standard deviation.

Abbreviations
TUG: Timed-Up-and-Go test; ASMM: Appendicular Skeletal Muscle Mass; 6MWT: 6-Minute Walking test.
